# Relationship between the non-HDLc-to-HDLc ratio and carotid plaques in a high stroke risk population: a cross-sectional study in China

**DOI:** 10.1186/s12944-020-01344-1

**Published:** 2020-07-13

**Authors:** Yan Liu, Zhenwen Zhang, Binlan Xia, Liping Wang, Hengzhong Zhang, Yan Zhu, Chao Liu, Bin Song

**Affiliations:** 1 Endocrine and Diabetes Center, Affiliated Hospital of Integrated Traditional Chinese and Western Medicine, Nanjing University of Chinese Medicine, 8 Huadian East Road, Nanjing, 210028 China; 2 Department of Endocrinology, Clinical Medical College, Yangzhou University, 98 Nantong West Road, Yangzhou, 225001 China; 3 Department of Ultrasonography, Clinical Medical College, Yangzhou University, 98 Nantong West Road, Yangzhou, 225001 China; 4 Department of Biobank, Clinical Medical College, Yangzhou University, 98 Nantong West Road, Yangzhou, 225001 China; 5 Department of Center of Health Management, Clinical Medical College, Yangzhou University, 98 Nantong West Road, Yangzhou, 225001 China

**Keywords:** Carotid plaques, Non-high-density lipoprotein cholesterol, High-density lipoprotein cholesterol, High stroke risk, Cross-sectional, Ratio, Atherosclerosis

## Abstract

**Background:**

Evidence on the association between the non-high-density lipoprotein cholesterol (non-HDLc)-to-high-density lipoprotein cholesterol (HDLc) ratio (non-HDLc/HDLc) and carotid plaques is still limited. This study aims to assess the relationship between the non-HDLc/HDLc and carotid plaques in a population with a high risk of stroke.

**Methods:**

A cross-sectional study based on the community was conducted in Yangzhou, China. Residents (no younger than 40 years old) underwent questionnaire interviews, physical examinations, and laboratory testing during 2013–2014. The subjects with a high risk of stroke were further selected (at least three of eight risk factors including hypertension, atrial fibrillation, type 2 diabetes mellitus, dyslipidaemia, smoking, lack of exercise, overweight, and family history of stroke) or a transient ischaemic attack (TIA) or stroke history. Carotid ultrasonography was then performed on the high stroke risk participants. Carotid plaque was defined as a focal carotid intima-media thickness (cIMT) ≥1.5 cm or a discrete structure protruding into the arterial lumen at least 50% of the surrounding cIMT. Logistic regression was employed to evaluate the relationship between the non-HDLc/HDLc and carotid plaques.

**Results:**

Overall, 839 subjects with a high risk of stroke were ultimately included in the analysis, and carotid plaques were identified in 341 (40.6%) of them. Participants in the highest non-HDLc/HDLc tertile group presented a higher proportion of carotid plaques than did those in the other two groups. After adjustment for other confounders, each unit increase in the non-HDLc/HDLc was significantly associated with carotid plaques (OR 1.55, 95%CI 1.28–1.88). In the subgroup analysis, the non-HDLc/HDLc was positively and significantly associated with the presence of carotid plaques in most subgroups. Additionally, the non-HDLc/HDLc interacted significantly with three stratification variables, including sex (OR 1.31 for males vs. OR 2.37 for females, *P* interaction = 0.016), exercise (OR 1.18 for subjects without lack of exercise vs. OR 1.99 for subjects with lack of exercise, *P* interaction = 0.004) and heart diseases (OR 1.40 for subjects without heart diseases vs. OR 3.12 for subjects with heart diseases, *P* interaction = 0.033).

**Conclusion:**

The non-HDLc/HDLc was positively associated with the presence of carotid plaques in a Chinese high stroke risk population. A prospective study or randomized clinical trial of lipid-lowering therapy in the Chinese population is needed to evaluate their causal relationship.

## Background

As one of the major atherosclerotic diseases, carotid atherosclerosis (CA) has become a leading cause of ischaemic stroke [1]. Reflecting the severity of lumen narrowing and irregular morphology of the carotid artery, carotid plaques, detected by non-invasive ultrasonography, could serve as a subclinical indicator of CA and provide early information to predict ischaemic stroke risk [2]. Therefore, it will be of great importance to prevent ischaemic stroke early by detecting and intervening in the risk factors for carotid plaques.

Previous investigations have indicated that the non-high-density lipoprotein cholesterol (non-HDLc)-to-high-density lipoprotein cholesterol (HDLc) ratio (non-HDLc/HDLc) is significantly associated with arterial stiffness [3], non-alcoholic fatty liver diseases [4, 5], chronic kidney disease [6], diabetes [7], metabolic syndrome [8, 9] and insulin resistance [9]. Among these, some scholars found that the non-HDLc/HDLc was superior to either non-HDLc or HDLc alone [3, 5]. In addition, previous studies have investigated relationships between serum lipid profiles (including non-HDLc and HDLc) and CA [10–15]. One study found that the non-HDLc/HDLc was positively associated with the carotid intima-media thickness (cIMT) [14], and another study even indicated that postmenopausal females with a higher non-HDLc/HDLc were more likely to have carotid plaques [15]. However, the relationship between the non-HDLc/HDLc and carotid plaques is still largely unexplored in different populations.

Therefore, the present cross-sectional investigation was carried out in a population with a high stroke risk in China to elucidate whether the non-HDLc/HDLc is independently related to the presence of carotid plaques.

## Methods

### Study population

The Stroke Screening and Intervention Program (SSIP), a community-based, observational study implemented by the National Stroke Prevention Committee of China since 2012, was designed to evaluate and intervene in the stroke risk factors in Chinese adults. As a part of the SSIP, details of the study design and population used in the present cross-sectional study were reported elsewhere [10, 16]. From among the initially recruited population, two communities (one from an urban location and the other from a rural location) were selected from November 2013 to March 2014 in Yangzhou (a city in eastern China), where most residents had lived for at least six months. A sample of 5529 inhabitants older than 40 years was randomly selected using stratified sampling by age and sex, based on the census results from the Chinese National Bureau of Statistics in 2010. In total, 5103 residents participated in this study, and complete baseline data were ultimately obtained from 4847 individuals.

High stroke risk participants were further selected to be analysed in this study, as only this population underwent carotid ultrasound measurement. The major inclusion criteria based on a holistic assessment of their risk factors for stroke were (1) hypertension, defined as elevated systolic blood pressure (SBP ≥ 140 mmHg) or diastolic blood pressure (DBP ≥ 90 mmHg) or use of antihypertensive drugs; (2) AF (atrial fibrillation), defined as an abnormal electrocardiogram suggesting atrial fibrillation; (3) type 2 diabetes mellitus, defined as elevated blood glucose (fasting ≥7.0 mmol/L or postprandial 2 h ≥ 11.1 mmol/L or glycosylated haemoglobin ≥6.5%), or treatment with oral antidiabetic medication or insulin; (4) dyslipidaemia, defined as triglyceride (TG) ≥ 1.70 mmol/L or total cholesterol (TC) ≥ 5.20 mmol/L or low-density lipoprotein cholesterol (LDLc) ≥ 3.36 mmol/L or HDLc ≤0.90 mmol/L or current treatment with antilipidaemic medication; (5) smoking, defined as former history or current status of smoking; (6) lack of exercise, defined as physical exercise less than 30 min each time or less than three times a week or exercise duration less than one year (agricultural or industrial labour was regarded as no lack of exercise); (7) overweight, defined as body mass index (BMI) ≥ 26 kg/m^2^; and (8) family history of stroke. Participants with at least three of the above eight risk features or history of a transient ischaemic attack (TIA) or stroke were considered members of the population with a high stroke risk. The major exclusion criteria were (1) pregnant woman or those who had recently delivered a child; (2) participants with renal insufficiency or other severe systemic illness; and (3) incomplete data collection without key parameters such as lipids or carotid plaques. The SSIP was conducted based on the Helsinki Declaration in 1975 and approved by the medical ethics committee of Jiangsu Subei People’s Hospital (serial number: 2013KY-049). All participants signed written informed consent prior to any specimen or data collection.

### Data collection

As previously described [10, 16], data on age, sex, region, education, employment status, smoking and exercise status, medical history and previous medication were collected by trained medical staff within a structured questionnaire. Physical examination was performed to measure height, weight, SBP and DBP. Overnight fasting blood samples were collected from each participant to measure fasting blood glucose (FBG), TG, TC, LDLc, HDLc, and homocysteine (HCY) levels with an AU6800 automatic chemistry analyser (Beckman Coulter, Brea, CA, USA) at the central laboratory of Jiangsu Subei People’s Hospital. Normal reference ranges were 3.89–6.11 mmol/L for FBG, 0.56–1.70 mmol/L for TG, 2.8–5.9 mmol/L for TC, 2.07–3.36 mmol/L for LDLc, 0.90–1.55 mmol/L for HDLc, and 0.0–15.0 μmol/L for HCY. The value of non-HDLc was obtained by subtracting HDLc from TC [11], and elevated HCY was defined as > 15.0 μmol/L.

### Carotid ultrasonography

Carotid ultrasonography was conducted by certified sonographers using a portable instrument (*LOGIC P5, 10.0 MHz,* GE Healthcare Inc.; Boston, MA, USA). Carotid plaques were detected in three locations, including the common and internal carotid arteries and carotid bifurcation. Longitudinal and transverse scanning were performed on the far and near walls of these sections. The distance between the media-adventitia and lumen-intima boundaries was quantified at plaque-free sections of the carotid artery and defined as cIMT. Carotid plaque was defined as a focal cIMT ≥1.5 cm or a discrete structure protruding into the arterial lumen more than 50 % from the surrounding cIMT, based on the American Society of Echocardiography [17].

### Statistical analysis

Normally distributed continuous variables are described as the mean ± standard deviation (SD) and were compared by one-way ANOVA, while non-normally distributed continuous variables are presented as the median (interquartile ranges) and were compared using the Kruskal–Wallis test. Categorical variables are summarized as counts with percentages and were compared using the chi-squared test or Fisher exact probability test. Subjects were also categorized into three groups in accordance with non-HDLc/HDLc tertiles. Logistic regression was employed to evaluate the correlation between the non-HDLc/HDLc (either as a continuous or categorical variable) and carotid plaques and calculate odds ratios (ORs) and 95% confidence intervals (CIs). For multiple regression analysis, collinearity among independent variables was first assessed to ensure that it was appropriate to include them in the same model. If variance inflation factors were no less than 5, collinearity was considered to exist, and such a variable was not included in the adjusted models. Finally, adjusted covariables included sex, age, region, education, employment status, BMI, SBP, DBP, FBG, TG, HCY, exercise and smoking status, family history of stroke, history of stroke and TIA, hypertension, type 2 diabetes, heart diseases and current antilipidaemic medication.

Subgroup analyses were performed based on some of the stroke risk factors and potential confounders mentioned above. For a continuous variable, it was first converted to a categorical variable according to the clinical or reference range cut-off point. Finally, subgroup analyses were conducted for the following variables: sex (male, female), age (< 65, ≥65 years), region (urban, rural), overweight (yes/no), lack of exercise (yes/no), smoking (yes/no), family history of stroke (yes/no), history of stroke (yes/no), heart diseases (yes/no), hypertension (yes/no), type 2 diabetes (yes/no), current antilipidaemic medication (yes/no), and high HCY (yes/no). The heterogeneity was also evaluated by incorporating two-factor interaction terms between the non-HDLc/HDLc (continuous) and included stratification variable, followed by the likelihood ratio test. All analyses were performed using Empower Stats (www.empowerstats.com, X&Y solutions, Inc. Boston, MA, USA) and R software version 3.4.3 (http://www.r-project.org). Statistical significance was evaluated by a two-sided test with *P* < 0.05.

## Results

### Participants’ baseline characteristics

**Fig. 1:**
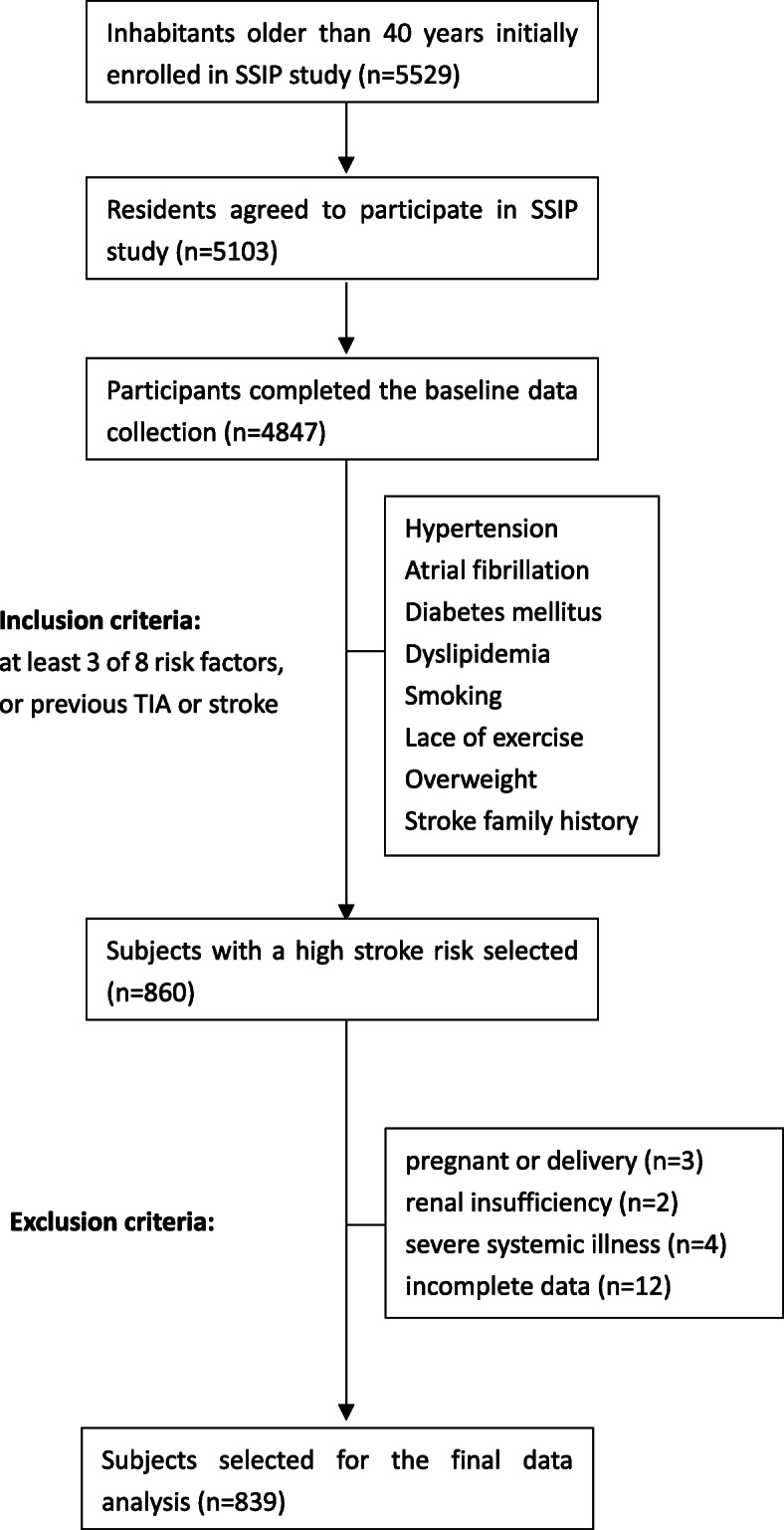
Flowchart of participants selection

For the data analysis, a total of 839 residents were ultimately enrolled in this study (Fig. 1 for a flow chart). The participants’ baseline characteristics were shown in Table 1 according to tertiles of the non-HDLc/HDLc. Generally, all selected participants had an average age of 63.6 ± 9.9 years old. Males accounted for 54.2% of the subjects, and carotid plaques were identified in 341 (40.6%) of the 839 high stroke risk participants. The current sample size of 839 subjects with 341 cases had 80% power to detect at least an odds ratio of the non-HDLc/HDLc on carotid plaques at a two-tailed *P* value of < 0.05. The average value of the non-HDLc/HDLc was 2.88 ± 1.05. Participants in the highest tertile group of the non-HDLc/HDLc (T3) were more likely to be male and had higher values of FBG, TG, TC, and LDLc, and higher education levels, and higher proportion of smoking and carotid plaques than those in the other groups. The opposite patterns were observed for age, HDLc and the proportion of urban regions. No statistically significant differences were detected in BMI, SBP, DBP, HCY, employment status, overweight, exercise status, family history of stroke, history of stroke and TIA, hypertension, type 2 diabetes, heart diseases, elevated HCY or current antilipidaemic medication among the different non-HDLc/HDLc groups (all *P* values > 0.05).

**Table 1:** Baseline characteristics of participants

Characteristic	non-HDLc/HDLc (tertile)			F/_**x**_2	***P***
	T1 (***n*** = 287)	T2 (***n*** = 286)	T3 (***n*** = 266)		
Male	137 (47.7)	148 (51.8)	170 (63.9)	15.629	< 0.001
Age (years)	64.8 ± 10.0	63.2 ± 9.4	62.7 ± 10.2	3.575	0.028
BMI (kg/m^2^)	25.54 ± 3.63	25.81 ± 2.99	26.05 ± 3.20	1.612	0.200
SBP (mmHg)	140.08 ± 15.55	140.91 ± 13.34	139.80 ± 16.01	0.411	0.663
DBP (mmHg)	86.87 ± 9.17	87.99 ± 8.78	87.38 ± 9.62	1.051	0.350
FBG (mmol/L)	6.27 ± 1.87	6.41 ± 2.03	6.84 ± 2.48	5.208	0.006
TG (mmol/L)	1.13 (0.84–1.42)	1.54 (1.19–2.00)	2.25 (1.60–3.00)	2.348	< 0.001
TC (mmol/L)	4.27 ± 0.82	4.78 ± 0.78	5.22 ± 0.92	88.790	< 0.001
LDLc (mmol/L)	2.14 ± 0.58	2.70 ± 0.67	2.93 ± 0.81	96.581	< 0.001
HDLc (mmol/L)	1.56 ± 0.35	1.25 ± 0.22	1.03 ± 0.19	287.603	< 0.001
non-HDLc (mmol/L)	2.71 ± 0.64	3.53 ± 0.59	4.19 ± 0.78	337.441	< 0.001
non-HDLc/HDLc	1.78 ± 0.41	2.84 ± 0.29	4.10 ± 0.61	1810.918	< 0.001
HCY (μmol/L)	9.0 (6.0–13.0)	9.5 (7.0–12.4)	9.3 (6.5–13.0)	2.350	0.422
Urban	179 (62.4)	147 (51.4)	102 (38.4)	31.908	< 0.001
Education				18.664	0.005
Primary school or under	125 (43.6)	101 (35.3)	81 (30.5)		
Middle or high school	132 (45.9)	157 (54.9)	152 (57.1)		
College or above	14 (4.9)	18 (6.3)	26 (9.8)		
Not reported	16 (5.6)	10 (3.5)	7 (2.6)		
Employment status				9.479	0.050
Unemployed	81 (28.2)	95 (33.2)	107 (40.2)		
Employed	169 (58.9)	158 (55.3)	135 (50.8)		
Not reported	37 (12.9)	33 (11.5)	24 (9.0)		
Overweight	131 (45.6)	132 (46.2)	137 (51.5)	2.303	0.316
Smoking	96 (33.5)	117 (40.9)	129 (48.5)	12.948	0.002
Lack of exercise	156 (54.4)	163 (57.0)	143 (53.8)	0.671	0.715
Family history of stroke	60 (20.9)	62 (21.7)	54 (20.3)	0.159	0.924
History of stroke	67 (23.3)	60 (21.0)	49 (18.4)	2.019	0.364
History of TIA	13 (4.5)	17 (5.9)	9 (3.4)	2.053	0.358
Heart diseases	79 (27.5)	65 (22.7)	60 (22.6)	2.447	0.294
Hypertension	260 (90.6)	248 (86.7)	227 (85.3)	3.827	0.148
Type 2 diabetes	81 (28.2)	83 (29.0)	92 (34.6)	3.092	0.213
Current antilipidaemic medication	31 (10.8)	26 (9.1)	32 (12.0)	1.273	0.529
High HCY	42 (14.6)	39 (13.6)	50 (18.8)	3.103	0.212
Carotid plaques	108 (37.6)	108 (37.8)	125 (47.0)	6.509	0.039

T1, non-HDLc/HDLc: 0.41–2.35; T2, non-HDLc/HDLc: 2.36–3.36; T3, non-HDLc/HDLc: 3.37–5.98. Continuous data are shown as the mean ± standard deviation or median (interquartile), and categorical data are shown as n (%). For categorical variables, *P* value was calculated by χ2 or Fisher’s exact test. For continuous variables, *P* value was calculated by one-way ANOVA or Kruskal–Wallis test

*BMI* body mass index, *SBP* systolic pressure, *DBP* diastolic pressure, *FBG* fasting blood glucose, *TG* triglyceride, *TC* total cholesterol, *LDLc* low-density lipoprotein cholesterol, *HDLc* high-density lipoprotein cholesterol, *non-HDLc* non-high-density lipoprotein cholesterol, *HCY* homocysteine

### Univariate analysis of carotid plaques

By univariate binary logistic regression with carotid plaques as the dependent variable, shown in Table 2, it could be observed that male sex, age, SBP, DBP, FBG, TC, LDLc, non-HDLc, the non-HDLc/HDLc, HCY, rural region, smoking, history of stroke, hypertension, and elevated HCY were positively correlated with carotid plaques. In contrast, lack of exercise was negatively correlated with carotid plaques. Additionally, BMI, TG, HDLc, education, employment status, overweight, stroke family history, TIA, heart diseases, type 2 diabetes and current antilipidaemic medication were not significantly associated with carotid plaques.

**Table 2:** Univariate analysis of carotid plaques

Variables	Statistics	OR (95%CI)	β	***P***
Sex				
Female	384 (45.8)	Ref		
Male	455 (54.2)	1.50 (1.13, 1.98)	0.402	0.005
Age (years)	63.6 ± 9.9	1.07 (1.05, 1.09)	0.068	< 0.001
BMI (kg/m^2^)	25.79 ± 3.29	0.97 (0.93, 1.01)	− 0.033	0.132
SBP (mmHg)	140.27 ± 14.98	1.02 (1.01, 1.03)	0.021	< 0.001
DBP (mmHg)	87.41 ± 9.19	1.02 (1.00, 1.03)	0.016	0.033
FBG (mmol/L)	6.50 ± 2.15	1.07 (1.00, 1.14)	0.069	0.035
TG (mmol/L)	1.53 (1.12–2.18)	0.95 (0.86, 1.05)	−0.048	0.339
TC (mmol/L)	4.74 ± 0.92	1.33 (1.14, 1.54)	0.282	< 0.001
LDLc (mmol/L)	2.58 ± 0.76	1.45 (1.20, 1.75)	0.371	< 0.001
HDLc (mmol/L)	1.29 ± 0.34	0.93 (0.62, 1.40)	−0.072	0.730
non-HDLc (mmol/L)	3.46 ± 0.90	1.36 (1.16, 1.59)	0.306	< 0.001
non-HDLc/HDLc	2.88 ± 1.05	1.23 (1.07, 1.40)	0.204	0.003
HCY (μmol/L)	9.0 (6.0–13.0)	1.06 (1.04, 1.09)	0.059	< 0.001
Region				
Urban	428 (51.0)	Ref		
Rural	411 (49.0)	1.46 (1.10, 1.92)	0.376	0.008
Education				
Primary school or under	307 (36.6)	Ref		
Middle or high school	441 (52.6)	0.78 (0.58, 1.04)	−0.253	0.094
College and above	58 (6.9)	1.11 (0.63, 1.95)	0.104	0.717
Not reported	33 (3.9)	0.73 (0.35, 1.53)	−0.317	0.403
Employment status				
Unemployed	283 (33.7)	Ref		
Employed	462 (55.1)	1.03 (0.76, 1.39)	0.026	0.866
Not reported	94 (11.2)	1.01 (0.63, 1.62)	0.006	0.981
Overweight				
No	439 (52.3)	Ref		
Yes	400 (47.7)	0.83 (0.63, 1.09)	−0.190	0.178
Lack of exercise				
No	377 (44.9)	Ref		
Yes	462 (55.1)	0.70 (0.53, 0.93)	−0.355	0.012
Smoking				
No	497 (59.2)	Ref		
Yes	342 (40.8)	1.57 (1.18, 2.07)	0.449	0.002
Family history of stroke				
No	663 (79.0)	Ref		
Yes	176 (21.0)	1.08 (0.77, 1.51)	0.073	0.670
History of stroke				
No	663 (79.0)	Ref		
Yes	176 (21.0)	1.67 (1.19, 2.33)	0.512	0.003
History of TIA				
No	800 (95.4)	Ref		
Yes	39 (4.7)	1.41 (0.74, 2.68)	0.344	0.295
Heart diseases				
No	635 (75.7)	Ref		
Yes	204 (24.3)	1.21 (0.88, 1.66)	0.189	0.246
Hypertension				
No	104 (12.4)	Ref		
Yes	735 (87.6)	2.13 (1.34, 3.38)	0.755	0.001
Type 2 diabetes				
No	583 (69.5)	Ref		
Yes	256 (30.5)	1.23 (0.91, 1.66)	0.207	0.172
Current antilipidaemic medication				
No	750 (89.4)	Ref		
Yes	89 (10.6)	1.49 (0.96, 2.32)	0.400	0.075
High HCY				
No	708 (84.4)	Ref		
Yes	131 (15.6)	2.40 (1.64, 3.51)	0.875	< 0.001

Continuous data are shown as the mean ± standard deviation or median (interquartile), and categorical data are shown as n (%)

*OR* odds ratio, *CI* confidence interval, *β* regression coefficient, *Ref* reference

### Associations of non-HDLc/HDLc and other crude lipid parameters with carotid plaques

Three logistic regression models were constructed to evaluate the independent effects of the non-HDLc/HDLc on carotid plaques (Table 3). In the unadjusted model, the non-HDLc/HDLc, considered as a continuous variable (per unit increase), was significantly associated with the presence of carotid plaques (OR 1.23, 95%CI 1.07–1.40). In the minimally adjusted model, as the non-HDLc/HDLc increased by one unit, the chance of having carotid plaques increased by 32% (OR 1.32, 95%CI 1.13–1.53). In the fully adjusted model, for each additional unit of the non-HDLc/HDLc, the chance of having carotid plaques increased by 55% (OR 1.55, 95%CI 1.28–1.88). For the purpose of sensitivity analysis, the continuous non-HDLc/HDLc was converted into a categorical variable (tertiles), and the *P* for trend of the non-HDLc/HDLc with carotid plaques in the unadjusted or adjusted model was consistent with the results when the non-HDLc/HDLc served as a continuous variable.

**Table 3:** Relationships between the non-HDLc/HDLc and carotid plaques

Variables	Participant without carotid plaque (n, %)	Participant with carotid plaque (n, %)	Unadjusted OR (95%CI)	***P***	Minimally adjusted OR (95%CI)	***P***	Fully adjusted OR (95%CI)	***P***
non-HDLc/HDLc	498	341	1.23 (1.07, 1.40)	0.003	1.32 (1.13, 1.53)	< 0.001	1.55 (1.28, 1.88)	< 0.001
non-HDLc/HDLc (tertile)								
T1	179 (35.9)	108 (31.7)	Ref		Ref		Ref	
T2	178 (35.7)	108 (31.7)	1.01 (0.72, 1.41)	0.974	1.12 (0.78, 1.62)	0.546	1.18 (0.81, 1.73)	0.393
T3	141 (28.3)	125 (36.7)	1.47 (1.05, 2.06)	0.026	1.65 (1.13, 2.41)	0.010	2.01 (1.29, 3.12)	0.002
*P* for trend				0.027		0.010		0.002

Minimally adjusted OR controlled for sex, age, region, education, employment status, BMI, exercise and smoking status, and family history of stroke

Fully adjusted OR controlled for sex, age, region, education, employment status, BMI, SBP, DBP, FBG, TG, HCY, exercise and smoking status, family history of stroke, history of stroke and TIA, hypertension, type 2 diabetes, heart diseases and current antilipidaemic medication

*OR* odds ratio, *CI* confidence interval, *Ref* reference

Additionally, as shown in Table 4, in the fully adjusted model, continuous non-HDLc (OR 1.73, 95%CI 1.42–2.11), TC (OR 1.57, 95%CI 1.31–1.89) and LDLc (OR 1.71, 95%CI 1.37–2.13) were significantly associated with carotid plaques, whereas neither HDLc or TG was not significantly associated with carotid plaques in either the unadjusted or adjusted models. In the mutually adjusted models (Additional file 1), both of the non-HDLc/HDLc ratio (OR 1.30, 95%CI 1.03–1.63) and non-HDLc (OR 1.80, 95%CI 1.10–2.95) were positively and significantly associated with the presence of carotid plaques even after adjusted with LDLc. However. LDLc was no longer significantly associated with carotid plaques when adjusted with non-HDLc (OR 0.94, 95%CI 0.54–1.62).

**Table 4:** Relationships between the crude lipid parameters and carotid plaques

Variables	Unadjusted OR (95%CI)	***P***	Minimally adjusted OR (95%CI)	***P***	Fully adjusted OR (95%CI)	***P***
non-HDLc (mmol/L)	1.36 (1.16, 1.59)	< 0.001	1.52 (1.27, 1.80)	< 0.001	1.73 (1.42, 2.11)	< 0.001 ^a^
HDLc (mmol/L)	0.93 (0.62, 1.40)	0.730	0.90 (0.57, 1.40)	0.634	0.90 (0.55, 1.47)	0.675 ^a^
TC (mmol/L)	1.33 (1.14, 1.54)	< 0.001	1.45 (1.23, 1.71)	< 0.001	1.57 (1.31, 1.89)	< 0.001 ^a^
LDLc (mmol/L)	1.45 (1.20, 1.75)	< 0.001	1.59 (1.29, 1.95)	< 0.001	1.71 (1.37, 2.13)	< 0.001 ^a^
TG (mmol/L)	0.95 (0.86, 1.05)	0.339	0.99 (0.90, 1.10)	0.863	0.97 (0.87, 1.09)	0.662 ^b^

Minimally adjusted OR controlled for sex, age, region, education, employment status, BMI, exercise and smoking status, and family history of stroke

Fully adjusted OR controlled for sex, age, region, education, employment status, BMI, SBP, DBP, FBG, HCY, exercise and smoking status, family history of stroke, history of stroke and TIA, hypertension, type 2 diabetes, heart diseases and current antilipidaemic medication plus TG ^a^ or LDLc ^b^

*OR* odds ratio, *CI* confidence interval, *Ref* reference

### Associations of the non-HDLc/HDLc with carotid plaques in prespecified and exploratory subgroups

**Fig. 2:**
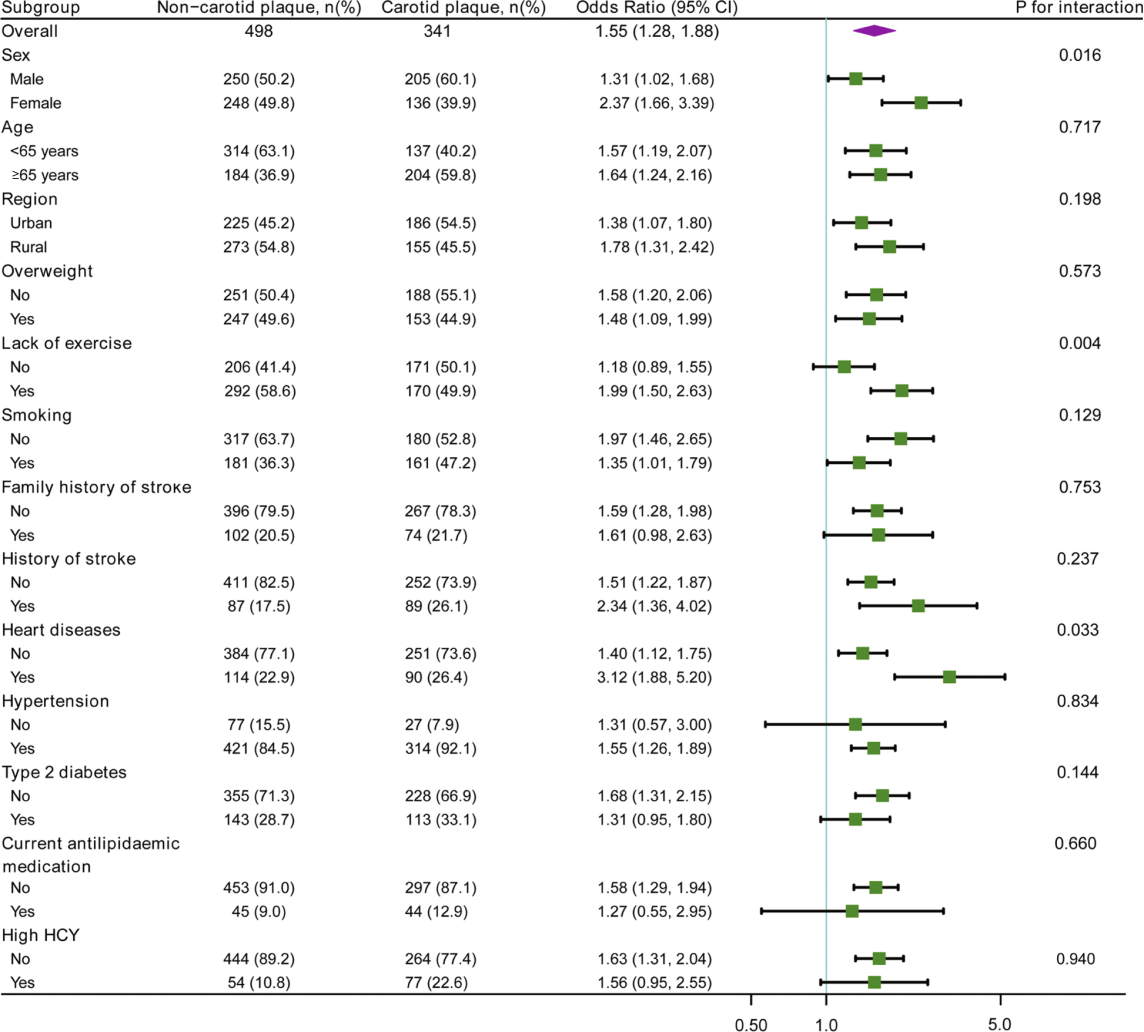
Associations of the non-HDLc /HDLc with carotid plaques in prespecified and exploratory subgroups. Odds ratios were adjusted for sex, age, region, education, employment status, BMI, SBP, DBP, FBG, TG, HCY, exercise and smoking status, family history of stroke, history of stroke and TIA, hypertension, type 2 diabetes, heart diseases and current antilipidaemic medication. In each case, the stratification variable itself in each subgroup analysis was not included in the adjusted model. *P* for interaction were from the likelihood ratio test

Subgroup analyses explored possible associations of the non-HDLc/HDLc (treated as a continuous variable) with carotid plaques among different stratification variables (Fig. 2). The associations between the non-HDLc/HDLc and carotid plaques were similar among the following subgroups: age, region, overweight, smoking, family history of stroke, history of stroke, hypertension, type 2 diabetes, current antilipidaemic medication, and high HCY (all *P* interaction > 0.05). Noticeably, the non-HDLc/HDLc interacted significantly with three stratification variables, including sex (OR 1.31, 95%CI 1.02–1.68 for males vs. OR 2.37, 95%CI 1.66–3.39 for females, *P* interaction = 0.016), exercise (OR 1.18, 95%CI 0.89–1.55 for subjects without lack of exercise vs. OR 1.99, 95%CI 1.50–2.63 for subjects with lack of exercise, *P* interaction = 0.004) and heart diseases (OR 1.40, 95%CI 1.12–1.75 for subjects without heart diseases vs. OR 3.12, 95%CI 1.88–5.20 for subjects with heart diseases, *P* interaction = 0.033).

## Discussion

In the present cross-sectional study based on the community population with a high risk of stroke in China, an independent, positive relationship was observed between the non-HDLc/HDLc – considered either as a continuous or categorical variable – and the presence of carotid plaques. Subjects had a 1.55-fold elevated chance of having carotid plaques with a per unit increase in the non-HDLc/HDLc after adjustment for other covariates, and this correlation was also found in most subgroups. The findings indicate that in a Chinese population with a high risk of stroke, an increased non-HDLc/HDLc is related to an elevated chance of having carotid plaques.

Non-HDLc represents atherogenic lipoproteins such as LDLc, very low-density lipoproteins, and small dense LDL (apolipoprotein B) [6], and the non-HDLc/HDLc ratio was detected to perform better than LDLc, HDLc or non-HDLc in estimating arterial stiffness [3] and non-alcoholic fatty liver disease [5]. However, there is limited evidence on the relationship between the non-HDLc/HDLc and carotid plaques. Qin et al. suggested that an increased non-HDLc/HDLc was significantly associated with elevated cIMT among Chinese individuals with metabolic syndrome [14]. The present study provides further evidence that the non-HDLc/HDLc was also positively correlated with the presence of carotid plaques. In addition, this study extends previous work on associations of the non-HDLc/HDLc with carotid plaques from postmenopausal middle-aged females [15] to the case of a high stoke risk population. Noticeably, the significant association between non-HDLc/HDLc with carotid plaques was mainly due to non-HDLc rather than HDLc, as HDLc did not show a significant influence in the present study. Furthermore, these findings from mutually adjusted models indicate that other components of non-HDLc beyond LDLc might have more contribution to carotid plaques than LDLc. In fact, previous studies have demonstrated that apolipoprotein B, but not LDLc, was associated with the progression of carotid plaques [18] or the future risk of coronary heart disease [19]. Therefore, the use of LDLc to assess cholesterol-related carotid plaques risk might underestimate future cardiovascular risk compared with small dense LDLc [18]. Although the concentration of small dense LDLc was not examined in this study, to a certain extent, the use of non-HDLc to assess carotid plaques appears to be superior to LDLc as non-HDLc components include small dense LDLc. Additionally, considering the non-significant effect of HDLc on carotid plaques in this study, the non-HDLc/HDLc ratio, as a clinically accessible indicator, might be more suitable to assess carotid plaques than LDLc.

In the univariate analysis, subjects with carotid plaques had higher values of age, SBP, DBP, FBG, and HCY and were more likely to be smokers, rural individuals and those with stroke history and hypertension, in agreement with the findings of previous studies [10, 20, 21]. Similar to the results in this study, Mi T et al. showed that men were more likely to suffer from carotid plaques in a high stroke risk population [10]. However, a higher chance of having carotid plaques was detected among women in a general population [22]. Additionally, the univariate analysis detected an unexpected phenomenon in which lack of exercise appeared to be a protective indicator against carotid plaques, which was inconsistent with the results of Mi T et al. [10]. Certainly, the protective effectiveness of lack of exercise disappeared in multiple analysis (data not shown). Nevertheless, the significant and positive association of the non-HDLc/HDLc with carotid plaques was stable after adjusting for as many potential confounders as possible, indicating that the non-HDLc/HDLc might be independently associated with the presence of carotid plaques. Subgroup analysis is known to be helpful for understanding the trend of the non-HDLc/HDLc with carotid plaques in different populations. Some stroke risk factors and potential confounders were taken into account as stratification variables. The findings from this study showed that an elevated non-HDLc/HDLc was still significantly and independently related to a higher chance of having carotid plaques in most subgroups, providing further evidence that overall, the non-HDLc/HDLc is a reliable parameter for evaluating the presence of carotid plaques in this high stroke risk population. Subgroup analyses also showed evidence that sex, exercise status and heart diseases affected the observed correlation of the non-HDLc/HDLc with the presence of carotid plaques. Higher ORs have been observed in women and subjects with a lack of exercise or heart diseases, which might deserve further research.

Previous studies presented inconsistent conclusions regarding the relationship between traditional lipid profiles and the presence of carotid plaques in different populations. A community-based study from China suggested that TC and LDLc, but not TG, were strongly associated with the presence of CA in a general population [12]. However, several studies have previously reported that TG was also positively and significantly associated with CA in different populations [10, 23–25]. In particular, a large Chinese study found that TG was independently associated with carotid plaques in a high-risk stroke population, whereas LDLc and TC were not significantly associated with carotid plaques [10]. Conversely, findings from this study showed that LDLc and TC, but not TG or HDLc, were independently related to the presence of carotid plaques. Therefore, this issue is still controversial in the literature. The obvious differences between this and previous studies might be caused by discrepancies in the population selection or sample size. Additionally, the effect of TG or HDLc may have been masked by the existence of other powerful stroke risk factors in this high stroke risk population.

As an ongoing inflammatory process, atherosclerosis could affect multiple vascular territories including carotid and coronary arteries. The concomitant presence of carotid and coronary plaques is a frequently encountered clinical problem, and they appear to share common risk factors including dyslipidemia, diabetes, hypertension, smoking and obesity [26–28]. Recent studies showed a positive role of dual lipid-lowering treatment in coronary atherosclerosis regression, and the extent of coronary plaque regression was also positively associated with non-HDLc reduction [29]. Additionally, a cohort study indicated that plasma proprotein convertase subtilisin/kexin type 9 (PCSK9) levels were positively correlated with 10-year progression of carotid atherosclerosis beyond LDLc [30]. These findings, when taken together with the results from this study, it would potentially implicative that dual lipid-lowering therapy may be also beneficial for carotid atherosclerosis regression.

### Study strength and limitations

This study has some strengths: (1) A strict statistical adjustment strategy has been used to minimize residual confounders, as this observational study is susceptible to potential confounding; (2) the target independent variable (non-HDLc/HDLc) was handled as either a continuous or categorical variable, which can reduce the contingency in the data analysis and enhance the robustness; (3) to make better use of data, effect modifier factor analysis was conducted and yielded stable conclusions in different subgroups in this study.

However, the findings of this study have several limitations. First, causal links between the non-HDLc/HDLc and carotid plaques cannot be established within a cross-sectional study design. Follow-up of these participants could help clarify this issue. Second, the subjects in this research are individuals with a high stroke risk. Therefore, universality or extrapolation is a certain deficiency in this study. The following study could extend this work to the general population. Third, although the multiple regression model adjusted for as many confounders as possible, residual confounding cannot be excluded due to the failure to collect other pivotal parameters, including apolipoprotein B, the values of blood pressure during different days, the course and control of diabetes, and alcohol consumption. Therefore, collecting such key parameters could be essential in the future. Finally, given original design constraint, the composition and classification of carotid plaques were not further examined during data collection. In addition, ultrasound was used to evaluate the presence of plaque in this study, which may be less reliable in comparison with the high-resolution magnetic resonance imaging (MRI) [31]. However, ultrasound is safe, easily accessible, and inexpensive. Nevertheless, the use of MRI to evaluate the composition and types of plaques would have provided a significant add on the scientific value.

## Conclusions

In the Chinese high stroke risk population based on the community, an elevated non-HDLc/HDLc was prominently correlated with the presence of carotid plaques. To assess the causal nature of the relationship, a prospective study or randomized clinical trial of lipid-lowering therapy is needed in the Chinese population.

## Supplementary information

**Additional file 1.**

## Data Availability

The datasets used in the present study are available from the corresponding author on reasonable request.

## Abbreviations

SSIP: Stroke Screening and Intervention Program
BMI: Body mass index
CA: Carotid atherosclerosis
cIMT: Carotid intima-media thickness
AF: Atrial fibrillation
SBP: Systolic blood pressure
DBP: Diastolic blood pressure
FBG: Fasting blood glucose
TG: Triglyceride
TC: Total cholesterol
non-HDLc: Non-high-density lipoprotein cholesterol
LDLc: Low-density lipoprotein cholesterol
HDLc: High-density lipoprotein cholesterol
HCY: Homocysteine
TIA: Transient ischemic attack
PCSK9: Proprotein convertase subtilisin/kexin type 9
MRI: Magnetic resonance imaging
SD: Standard deviation
Ref: Reference
OR: Odds ratio
CI: Confidence interval
