# Supplementary material for: Relationship between the non-HDLc-to-HDLc ratio and carotid plaques in a high stroke risk population: a cross-sectional study in China
Source: Lipids Health Dis. 2020 Jul 13;19:168. doi: 10.1186/s12944-020-01344-1 (PMC7359500; doi:10.1186/s12944-020-01344-1)
Supplement: Supplementary file 1 — Additional file 1. [file 12944_2020_1344_MOESM1_ESM.docx]

**Table S1.** Relationships between different lipid parameters and carotid plaques

| **Variables** | **Fully adjusted OR**  **(95%CI)** | ***P*** | **Mutually adjusted OR**  **(95%CI)** | ***P*** |
| --- | --- | --- | --- | --- |
| non-HDLc/HDLc | 1.55 (1.28, 1.88) | <0.001 | 1.30 (1.03, 1.63) | 0.025 ^a^ |
| non-HDLc (mmol/L) | 1.73 (1.42, 2.11) | <0.001 | 1.80 (1.10, 2.95) | 0.020 ^a^ |
| LDLc (mmol/L) | 1.71 (1.37, 2.13) | <0.001 | 0.94 (0.54, 1.62) | 0.815 ^b^ |

Fully adjusted OR controlled for sex, age, region, education, employment status, BMI, SBP, DBP, FBG, HCY, exercise and smoking status, family history of stroke, history of stroke and TIA, hypertension, type 2 diabetes, heart diseases and current antilipidaemic medicatio

Mutually adjusted OR controlled for sex, age, region, education, employment status, BMI, SBP, DBP, FBG, HCY, exercise and smoking status, family history of stroke, history of stroke and TIA, hypertension, type 2 diabetes, heart diseases and current antilipidaemic medication plus LDLc ^a^ or non-HDLc ^b^

*OR* odds ratio, *CI* confidence interval, *Ref* reference
